# Absence of lower genital tract lesions among women of reproductive age infected with *Schistosoma mansoni*: A cross-sectional study using a colposcope in Western Kenya

**DOI:** 10.1371/journal.pntd.0010473

**Published:** 2022-07-08

**Authors:** Huldah C. Sang, Pauline N. M. Mwinzi, Maurice R. Odiere, Isaac Onkanga, Fredrick Rawago, Pavitra Pillay, Eyrun Floerecke Kjetland

**Affiliations:** 1 Neglected Tropical Diseases Unit, Centre for Global Health Research, Kenya Medical Research Institute, Kisumu, Kenya; 2 Department of Biomedical and Clinical Technology, Faculty of Health Sciences, Durban University of Technology, Durban, South Africa; 3 Norwegian Centre for Imported and Tropical Diseases, Department of Infectious Diseases Ullevaal, Oslo University Hospital, Oslo, Norway; 4 Discipline of Public Health, Nelson Mandela School of Medicine, University of KwaZulu-Natal, Durban, South Africa; Ministère de la Santé Publique et de la Lutte contre les Endémies, NIGER

## Abstract

**Background:**

Female genital schistosomiasis (FGS) constitutes four different lesions known to be caused by *Schistosoma haematobium* ova deposited in the genital tract. *Schistosoma mansoni* ova may also be found in the genital tract. However, it is not known if *S*. *mansoni* causes lower genital tract lesions characteristic of FGS.

**Methodology:**

This study was conducted in 8 villages along the shores of Lake Victoria, western Kenya. Stool and urine samples, collected from women of reproductive age on three consecutive days, were analysed for *S*. *mansoni* and *S*. *haematobium* infection. *S*. *mansoni* positive and *S*. *haematobium* negative willing participants, aged 18–50 years were invited to answer a questionnaire (demographics, symptoms), undergo a gynaecological examination and cytology specimen collection by an FGS expert.

**Principal findings:**

Gynaecologic investigations were conducted in 147 *S*. *mansoni*-positive women who had a mean infection intensity of 253.3 epg (95% CI: 194.8–311.9 epg). Nearly 90% of them used Lake Victoria as their main water source. None were found to have cervicovaginal grainy sandy patches or rubbery papules. Homogenous yellow patches were found in 12/147 (8.2%) women. Women with homogenous yellow patches were significantly older (47 years) than the rest (34 years, p = 0.001). No association was found between intensity of *S*. *mansoni* infection and homogenous yellow patches (p = 0.70) or abnormal blood vessels (p = 0.14). *S*. *mansoni* infection intensity was not associated with genital itch, bloody or malodorous vaginal discharge.

**Conclusion:**

*S*. *mansoni* infection was neither associated with lower genital tract lesions nor symptoms typically found in women with FGS.

## Author summary

Women who have fresh water contact in rivers or lakes may get infected with the waterborne parasites, that can cause Bilharzia, also called schistosomiasis. In Africa, *Schistosoma (S*.*) haematobium* is associated with HIV acquisition, genital tract lesions, bloody and malodorous discharge. There is also decreased clearance of human papillomavirus (HPV) in women with genital schistosomiasis. There are occasional case reports showing eggs of *S*. *mansoni* (intestinal schistosomiasis) in the genitals. However, no study has systematically looked for the genital tract lesions of *S*. *mansoni* and the impact on gynaecological health has never been explored. We sought to explore if *S*. *mansoni* causes the lower genital tract genital lesions. In women living near Lake Victoria, Kenya, we included 147 women who had *S*. *mansoni* but not *S*. *haematobium* infection. We found that the few women who had signs of sequelae of schistosomiasis infection in the genitals, so-called homogenous yellow patches had all potentially been exposed to *S*. *haematobium* in their youth. In this study population we found no lower genital tract lesions or genital symptoms to be associated with *S*. *mansoni*.

## Introduction

Schistosomiasis is endemic in sub-Saharan Africa where close to 205 million people are infected and many more are at risk of infection [1]. The most vulnerable are children and women who frequently come into contact with snail-infested water while playing, or in the course of their daily chores [2]. Despite current treatment efforts with mass drug administration using praziquantel, re-infections and chronic infection occur leading to morbidity that may last for decades, including Female Genital Schistosomiasis (FGS) [3–5].

All the human *Schistosoma* species may deposit ova in the genitals, sometimes denoted “ectopic schistosomiasis”. *S*. *mansoni* ova, usually located in the lower gastrointestinal tract, are sometimes also found as far as the urinary tract and it is hypothesized that ova travel from the intestinal tract to the genital organs through blood vessel anastomoses in the pelvis [6,7]. However, lower genital tract lesions have almost exclusively been reported from *S*. *haematobium* infected women [8–14]. FGS affects an estimated 56 million women worldwide [15].

Kenya is endemic for both the intestinal form (caused by *S*. *mansoni*) and the urogenital form (caused by *S*. *haematobium*) of schistosomiasis, with an estimate of approximately 9 million people infected and approximately 17.4 million at risk [16]. *S*. *haematobium* infection manifests itself with bloody urine, pain on urination, and higher risk of bladder cancer, genital symptoms and lesions [13,17]. The lower genital tract manifestations in women are grainy sandy patches, homogenous yellow patches, rubbery papules, abnormal blood vessels and mucosal bleeding of the surfaces, both in the vagina wall and on the cervix [18]. FGS is associated with decreased fertility, ectopic pregnancies, abdomino-pelvic pain, genital itch, dyspareunia, foul smelling discharge, and has been found to be associated with HIV [19–23].

There are a number of case reports of *S*. *mansoni* ova in the genital tract and it has been hypothesized that it could lead to similar lower genital tract morbidity [24–26]. However, only a paucity of studies have explored this. A study in Brazil, where only *S*. *mansoni* is endemic, found no genital tract morbidity due to schistosomiasis [27], but *S*. *mansoni* infection intensity was low and participants had received repeated praziquantel treatment. A second study by Downs and others in an area endemic for both *S*. *haematobium* and *S*. *mansoni* in Tanzania reported that 5% of the women had *Schistosoma* ova in cytology smears, but the authors did not differentiate the cases according to *Schistosoma* species and genitalia were not inspected for lesions [28]. Interestingly, the prevalence of HIV was higher in the *S*. *mansoni* villages than in the *S*. *haematobium* villages [28]. A qualitative study in western Kenya found that genital health problems were reported among women exposed to *S*. *mansoni*, but no diagnostic tests were done and there was no control group [29]. Cunin *et al* found that people with very high infection intensity of dual infection with *S*. *mansoni* and *S. haematobium* made it more likely to find *S*. *mansoni* ova in urine, possibly as a result of “worm over-crowding” [7].

HIV/AIDS is a leading cause of death in Kenya and is disproportionately high in Nyanza, western Kenya along the shores of Lake Victoria [30,31], an area that also has a high prevalence of *S*. *mansoni* [32–35]. Based on these observations and the hypothesis that *S*. *mansoni* may also be a risk factor for HIV [28], we sought to explore the prevalence of FGS in a *S*. *mansoni*-endemic area, investigate if *S*. *mansoni* causes lesions in the lower genital tract, and characterize the gynaecological symptoms in women with *S*. *mansoni* infection.

## Methods

### Ethics statement

Approval for the study was obtained from the Kenya Medical Research Institute (KEMRI) Scientific Steering Committees as well as the KEMRI National Ethical Review Committee (KEMRI SSC # 2937). Consent documents and participation information were approved and provided to potential study participants in English or the local language, Dholuo. The local authorities and health professionals were informed about the study. Informed written consent was obtained from each participant. A unique coded identification number was assigned to each subject and was used for sample tracking. All identifiers were delinked from data for the analysis. Privacy and confidentiality were strictly maintained. The risks of the study were minimal, with only the temporary discomfort and brief embarrassment associated with the gynaecological examination. All participants were treated with 40 mg/kg praziquantel (PZQ) and those infected with soil-transmitted helminths (STHs) were treated with 400 mg albendazole. In addition, treatment was provided in accordance with the Kenyan syndromic management protocol for sexually transmitted infections (STI). Results and treatment were delivered privately to the participant by a local professional nurse immediately after the study procedures.

### Study sites

**Fig 1:**
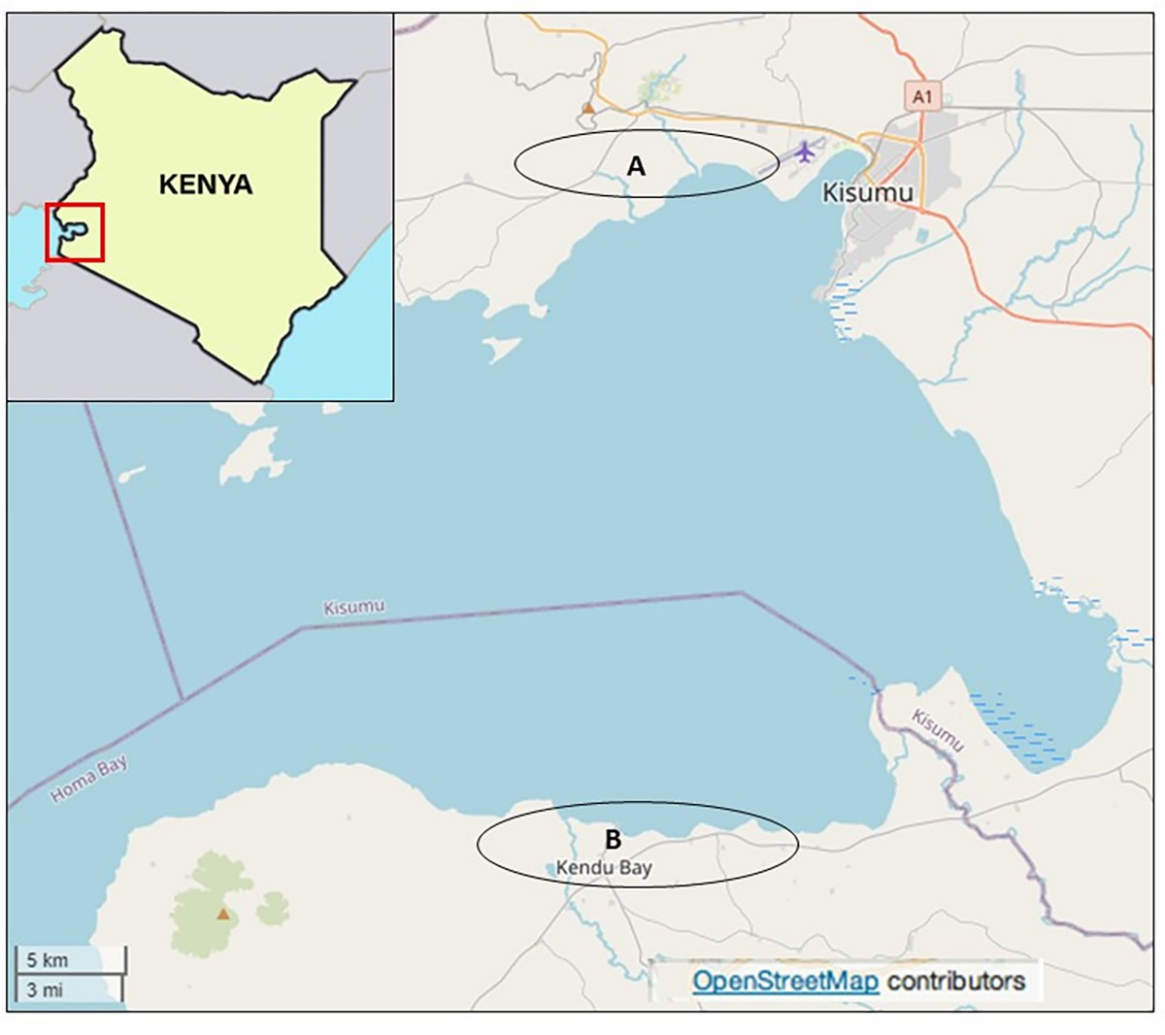
Study sites in western Kenya. (A) Kisumu West and (B) Rachuonyo North sub-counties, https://opensource.com/.

The study was conducted in Rachuonyo North and Kisumu West sub-counties (Fig 1), western Kenya in November 2015. The area is characterized by a modified equatorial climate. It is generally warm and humid with the long rains falling from March to May and the short rains between August and November/December, with an average annual temperature of 25°C. In this study, we focused on villages that are within a 5 km distance from Lake Victoria, where *S*. *mansoni* prevalence is the highest [36]. Together with other sub-counties bordering the lake, the region has the highest prevalence of HIV/AIDS (15.1%) in Kenya amongst persons aged 15–64 years [30]. The exact HIV prevalence in the selected communities was not known, however the overall prevalence of HIV in Kisumu was 16.3% [37]. The chief mode of HIV transmission is thought to be via heterosexual intercourse [38,39].

### Study population

The majority of the population consisted of the Luo ethnic community who were at risk of infection with *S*. *mansoni* through their occupational or recreational activities. This cross-sectional study focused on 18–50 year old women. At the time of the study, mass drug administration for schistosomiasis control was unavailable for adults in this endemic area.

**Fig 2:**
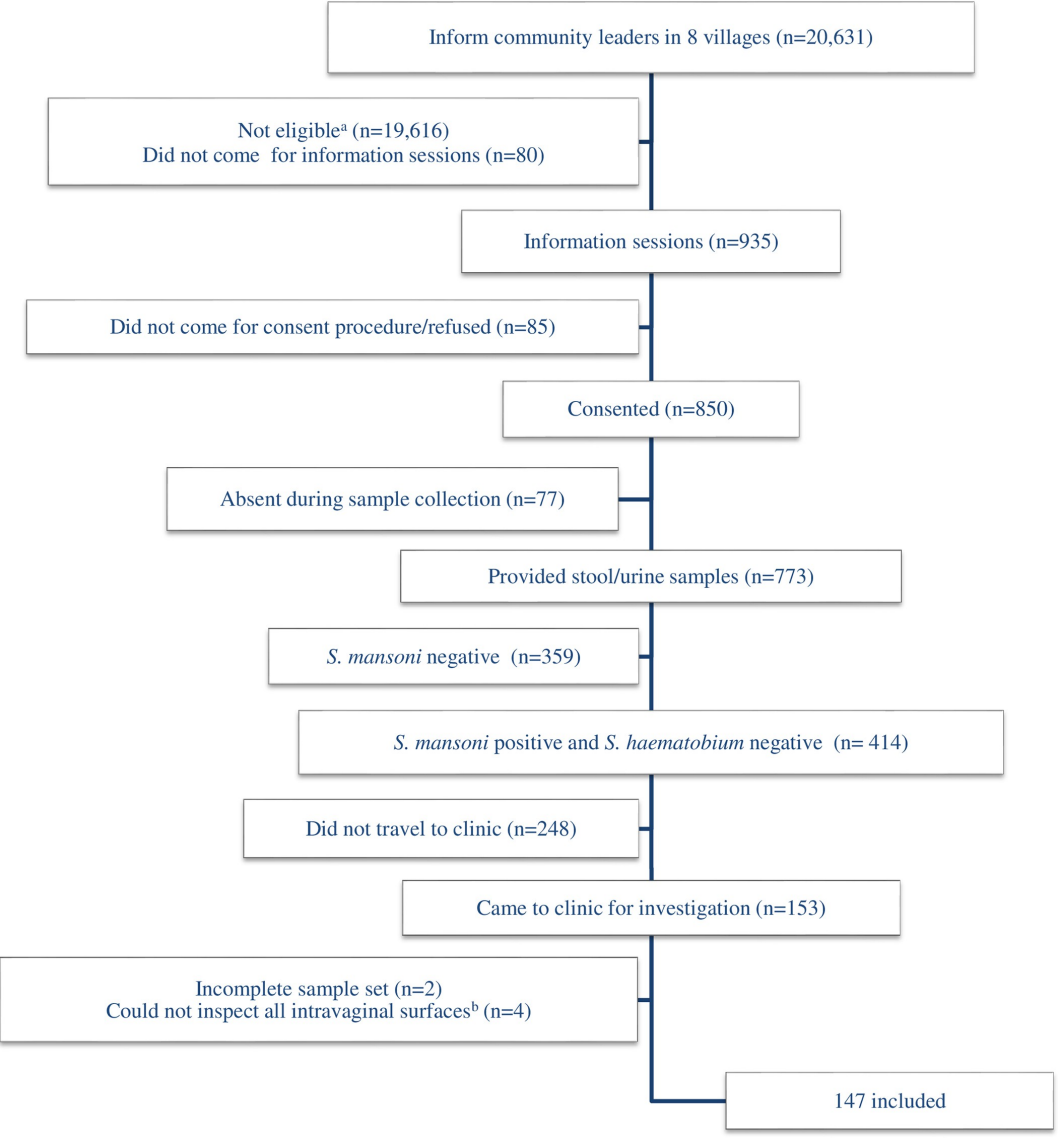
Participant recruitment. ^a^Males, children, outside age range. ^b^Cervix, fornices, lateral, anterior and/or posterior vaginal walls.

Women were approached at home by a community health worker to introduce the study and invite potential participants on a specific date to a specified health facility in their community. The investigators held information meetings and sought consent. Women who were not observably ill, were willing to take part in the study, and to provide the requested samples (stool, urine, blood, and intra-vaginal examination) were invited to participate. Only women who were *S*. *mansoni* egg-positive and *S*. *haematobium* egg-negative were invited for gynaecological examination. Fig 2 provides an overview of the participant recruitment process.

### Laboratory investigations

Stool and urine samples from three consecutive days were collected at the health facility, transported to the KEMRI laboratory and investigated microscopically for *S*. *mansoni* and *S*. *haematobium* eggs. Two Kato-Katz smears were prepared from each stool sample and read by trained microscopists. Eggs were enumerated to determine eggs per gram of faeces (epg) and the presence or absence of the STHs (*Ascaris lumbricoides*, *Trichuris trichiura*, hookworm) was noted. Intensity of infection for schistosomiasis was calculated based on arithmetic mean egg counts and categorized according to the WHO classification as negative for no detectable eggs; light *S*. *mansoni* infection for 1–99 epg; moderate for 100–399 epg and ≥400 epg for heavy [40]. Urine samples were thoroughly mixed and a 10 ml aliquot of urine filtered through 12 micrometres, 13 millimetres Polycarbonate (PCTE) membrane filters (Sterlitech; Kent, WA). The filter paper was then placed on a labelled slide and a drop of Lugol’s solution added. The slides were then examined under a microscope for *S*. *haematobium* and *S*. *mansoni* eggs [40].

### Questionnaires and clinical examinations

A questionnaire on water contact behaviour, reproductive history, water sources, water exposure, genital and abdominal symptoms was administered individually to *S*. *mansoni* egg-positive/*S*. *haematobium* egg-negative participants in the local language (Dholuo) prior to gynaecological examination. The clinician performing the exams was blinded to the childhood origin of the women and the intensity of *S*. *mansoni* infection. Examination was commenced by cervico-vaginal lavage. Saline (10 ml) was sprayed on the vaginal wall and cervix twice, whereupon it was drawn back into a syringe and deposited into four tubes. This was followed by photocolposcopic examination (Leisegang Photocolposcope, Germany, Magnifications 7.5; 15; 30) using an autoclaved metal speculum after which Pap (Papanicolau) smears were collected from all consenting women [41]. The cervix, the fornices, the entire vaginal wall and vulval surfaces were inspected section by section according to a predefined protocol [41]. Acetic acid and/or iodine application for colposcopic examination was always done last. The grainy sandy patch diagnosis was defined as observing grains approximately 0.05 mm by 0.2 mm long, shaped as minuscule rice grains, appearing singly or in clusters of up to 300 grains [42]. The homogeneous yellow patches were defined as sandy looking areas with no visible grains when using the x15 magnification setting on the colposcope. Rubbery papules were defined as papulous lesions, firm as hard rubber, that had only been seen in Madagascan women by the same clinician who had previously investigated women for FGS in four Southern African countries [43]. Abnormal blood vessels were defined as pathological convoluted (cork-screw), reticular, circular and/ or branched, uneven-calibre blood vessels visible (by x15 magnification) on the mucosal surface. Contact bleeding was defined as fresh blood originating from the mucosal surface. Pre-contact bleeding was defined as darkened blood on the mucosal surface in the absence of recent or present menstruation. A polyp was defined as a single, smooth pedunculated mass originating from the endocervix or from the mucosal surface. Leucoplakia was defined as white plaque on the mucosal surface, visible with or without acetic acid. Papilloma was defined as a sessile mass either on the mucosa or vulva, whitish in colour, often with a cauliflower appearance.

### Data management and statistical analyses

Data were entered into Excel, and analysed using SPSS version 12 and Graphpad Prism 5. Prevalence of *S*. *mansoni* and soil-transmitted helminths are presented for all who underwent gynaecological examination. *S*. *mansoni* data is also presented for those who submitted stool but did not attend the research clinic. Univariate and multivariate analyses were used to determine associations between the clinical manifestations and the intensity of *S*. *mansoni* infection. P values < 0.05 were considered statistically significant.

## Results

Out of the 773 women who provided stool samples, 414 (53.6%) had *S*. *mansoni* infection with a mean intensity of 185 epg (95%CI: 155–216 epg). A total of 72 women from Kisumu West and 79 from Rachuonyo North sub-counties came for gynaecological investigations. We included 147 women as 2 had recently delivered a baby and 2 had undergone hysterectomies. The mean age of menarche was 14 years (SD = 1.96).

Among the 147 women who underwent gynaecological examinations, the mean intensity of *S*. *mansoni* infection was 253 epg (95% CI: 195–312 epg). Table 1 shows that almost all these women had contact with Lake Victoria waters at least once per day, more than 90% lived less than 900 metres from the lake, and most used the lake as their main water source for domestic chores. Table 2 shows that more than half of the included population had heavy or moderate infections. The prevalence of the soil-transmitted helminths was 4.5% (95%CI: 3.3–6.3) for *T*. *trichiura*, 2.2% (95%CI: 1.4–3.5) for *A*. *lumbricoides*, and 0.3% (95%CI: 0.01–1) for hookworm respectively. No *S*. *mansoni* eggs were detected in the urine of any of the participants. None of the women had *Schistosoma* ova in their Pap smears.

**Table 1:** Characteristics of 147 women in *Schistosoma mansoni* endemic villages near Lake Victoria, Kenya.

*Variable*	*Statistic*	%
*Age (years)*		
18–19	5/147	3.4
20–24	29/147	19.7
25–29	20/147	13.6
30–39	45/147	30.6
40 and above	48/147	32.7
***Number of children***		
Infertile	2/147	1.4
1–3	69/147	46.9
4 and more	76/147	51.7
***Distance between home and Lake Victoria (km)***		
0–0.9	136/147	92.5
1–1.9	6/147	4.1
2–2.9	2/147	1.4
> 3	3/147	2.0
***Main water source***		
Lake Victoria	131/147	89.1
River	13/147	8.8
Borehole	3/147	2.0
***Frequency of contact with Lake Victoria (times/week)***		
None	3/147	2.0
1 to 7	83/147	56.4
8–27	40/147	27.2
28–41	14/147	9.5
42–56	7/147	4.8
***Main source of income***		
Farming	34/147	23.1
Fish trade	35/147	23.8
Housewife	21/147	14.3
Other	57/147	38.8

**Table 2:** Prevalence and intensity of *Schistosoma mansoni* infection in included and non-included women of reproductive age in Kisumu West and Rachuonyo North sub-Counties.

Sub-County	Category	N	Prevalence (CI)^1^	Intensity prevalence (%) Light Moderate Heavy			Infection intensity (epg)^2^ (CI)^1^
Kisumu West	Screened	262	45.8 (39.9–51.9)	54.2	27.5	18.3	243.3 (173.3–313.2)
	Included	70	100^c^ (93.7–100)	48.6	28.6	22.9	311.1 (201.1–421.1)
Rachuonyo North	Screened	511	57.5 (53.2–61.8)	61.2	27.5	11.2	161.6 (129.5–193.7)
	Included	77	100^c^ (94.3–100)	37.7	49.4	12.9	200.8 (150.8–250.9)
Overall	Screened	773	53.6 (50–57)	59.2	27.5	13.3	185.3 (154.7–215.8)
	Included	147	100^c^ (96.9–100)	42.8	39.5	17.7	253.3 (194.8–311.9)

^a^95% Confidence Interval.

^b^Arithmetic mean infection intensity of eggs found in stool.

^c^All included for gynae examination were *S*. *mansoni* positive

### Gynaecological examinations

**Fig 3:**
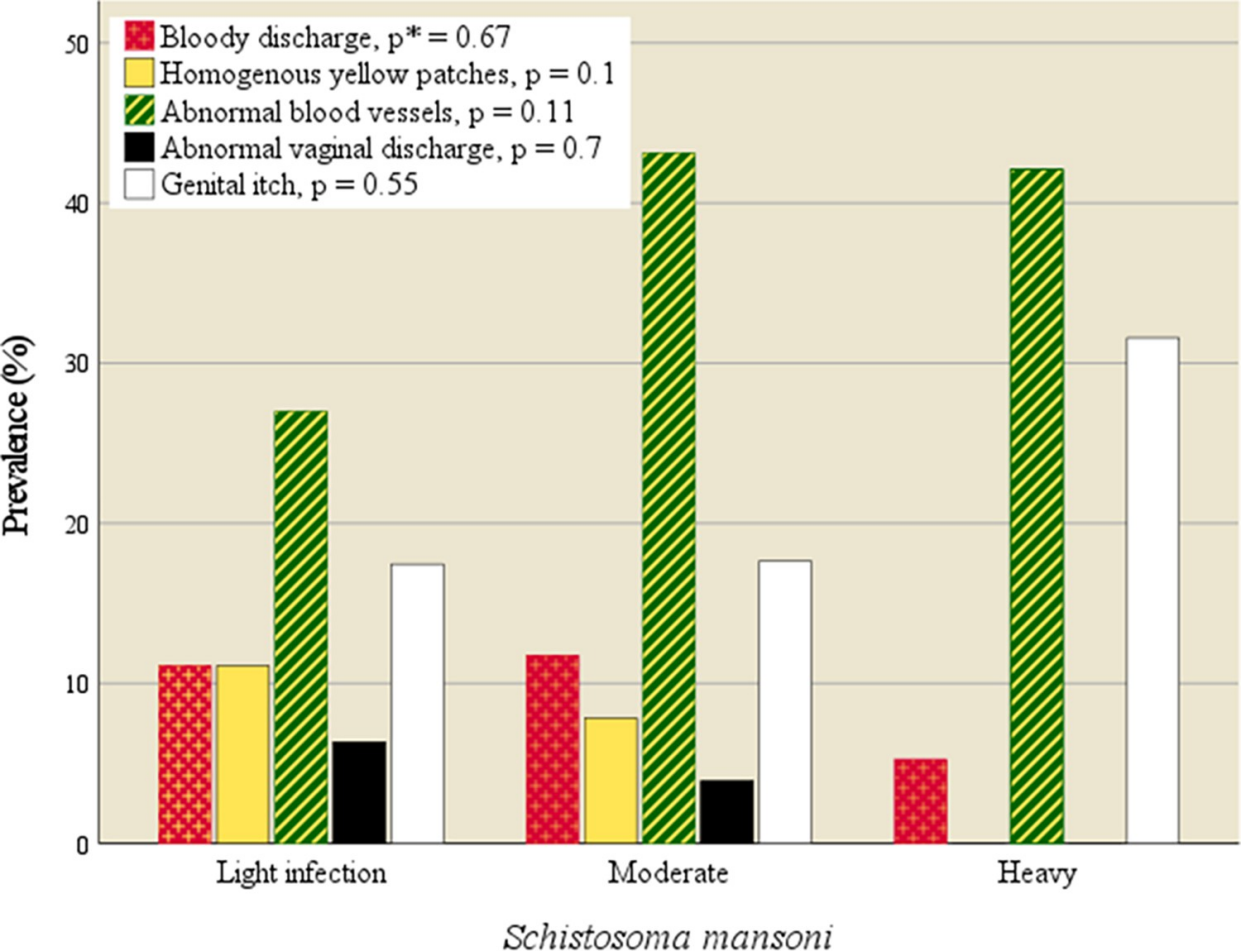
Genital symptoms and findings by *S*. *mansoni* intensity. No significant association between symptoms and intensity of *S*. *mansoni*. *Likelihood ratio p-value.

Homogenous yellow patches were found in 12/147 (8.2%) of the study population. No participants were found to have grainy sandy patches or rubbery papules. The women with homogenous yellow patches were significantly older than the remaining population (OR 1.1, 95% CI: 1.0–1.2, p = 0.001), mean age 47 years (SD 11.2) versus 34 years (SD 10.7). Homogenous yellow patches were not associated with the intensity of *S*. *mansoni* infection (age-Adjusted Odds Ratio (AOR) 0.6, 95% CI: 0.1–6.7, p = 0.70) (Fig 3). Likewise, having abnormal blood vessels on the mucosal surface (49/147, 33%), was not associated with the intensity of *S*. *mansoni* infection (AOR 1.0, 95% CI: 1.0–1.0, p = 0.14). In 7 of the 147 women (4.8%), not all the surfaces of the fornices could be seen.

### Symptoms

More than half of the population had genital symptoms (58%). *S*. *mansoni* intensity was not associated with higher prevalence of abnormal vaginal discharge (AOR 0.8, 95% CI: 0.1–6.7, p = 0.81), bloody discharge (AOR 0.2, 95% CI: 0.1–0.8, p = 0.21), or genital itch (AOR 0.8, 95% CI: 0.2–3.2, p = 0.79), as shown in Fig 3. Homogenous yellow patches (n = 12) were not associated with any of the symptoms (p >0.39).

### Potential *S*. *haematobium* exposure

In a selection of 140 women who were fully investigated gynecologically (all surfaces seen), 16 (11.4%) said they had grown up “far away” from Lake Victoria, although the exact distance could not be determined. They also indicated that they used other water sources in their childhood, such as rivers. These women had significantly more homogenous yellow patches (OR 5.6, 95% CI 1.4–21.8, p = 0.014). The age-adjusted odds ratio (AOR) was 3.9 (95% CI: 0.92–16.9, p = 0.065). Childhood residence or river contact did not influence the presence of abnormal blood vessels (p > 0.8).

## Discussion

In an area highly endemic for *Schistosoma mansoni*, we found that *S*. *mansoni* infection was not associated with any of the FGS lesions [18]. To our knowledge, this is the first study in women with *S*. *mansoni* where the antero-posterior and lateral vaginal surfaces, fornices and cervix were all inspected by an FGS expert, using a colposcope [14]. We are therefore confident we would have detected FGS, if present. Underpinning this, there was no increase in bloody discharge, abnormal vaginal discharge or lesions with heavy intensity of *S*. *mansoni* infection.

Previous studies have shown there is a strong association between *S*. *haematobium* ova in urine or genitals and four different lesion types [18,41]. A small proportion of women were found to have homogenous yellow patches, however no grainy sandy patches or rubbery papules were seen in this *S*. *mansoni* endemic area [42]. Although none of our patients had detectable *S*. *haematobium* (in three urines) many reported to have had river contact “elsewhere” in their youth or childhood. Urine egg excretion is known to decrease with age and our study population may have ceased to excrete *S*. *haematobium* ova [44,45]. Furthermore, in *S*. *haematobium* endemic areas, it is worth noting that, even in the absence of excretion of ova in urine, 23–41% of women have one of the typical FGS lesions and ova in the genitals [41,46]. We cannot preclude that homogenous yellow patches are due to long-standing or prior *S*. *haematobium* infection, they may be a sign of “aged” FGS lesions [47].

There is often a wide overlap in the geographic distribution of *S*. *haematobium* and *S*. *mansoni* in sub-Saharan Africa, making it difficult for single species incrimination [48]. In the absence of detectable ova in urine, other hard-to-achieve techniques such as species differentiation in histological specimens may be indicated or *S*. *haematobium*-specific PCR in cervicovaginal lavage or swabs [8,43,49–52]. While we did not observe any *S*. *mansoni* eggs in urine, we cannot rule out the possibility that homogenous yellow patches may have been induced by a tendency of schistosomes to shift their intravascular positions late in chronic infection as occurs with liver fibrosis when stool ova excretion may be low or there may be abnormal migration patterns of adult worms triggered by “worm crowding” [7,53–55]. In this population, however, nothing was found to indicate genital deposition *S*. *mansoni*ova and ensuing inflammation on the cervico-vaginal or vulval surfaces.

There is an increasing interest in the gender-specific manifestations of urogenital schistosomiasis with a growing appreciation of its multiple nexus as a chronic risk for HIV transmission. *S*. *mansoni* has been found in surgical specimens in Puerto Rico by Arean and others in the Fallopian tubes (18 cases), ovaries (10 cases); uterus or vulva (1 case), cervix (6 cases), and other case reports have also shown this [10,56–62]. However, most of these cases of *S*. *mansoni* were incidental findings in conjunction with malignancies, cervicitis or teratoma. In the cases with cervicitis differential diagnostic tests were not performed and hence *S*. *mansoni* cannot be ruled out as a cause.

FGS is a neglected disease and has previously been overlooked as a sexual and reproductive health rights issue [63,64], including its possible link with cervical cancer, HIV, and also mental health and social consequences [65,66]. *S*. *haematobium* infection is associated with abnormal vaginal discharge and bloody discharge, indicating intravaginal lesions or inflammation [13,21,67]. While *S*. *mansoni* ova can also be found in the genital tract, no study, including this one, has shown surface lesions of the intravaginal mucosa caused by *S*. *mansoni*. This study did not include *S*. *mansoni* negative women and we did not perform analyses for other diseases such as sexually transmitted diseases or endometriosis. However, the lack of increased number or extent of cervicovaginal lesions and symptoms in women with heavy intensity *S*. *mansoni* infections suggests that symptoms and findings were independent of *S*. *mansoni*. Furthermore, the same colposcope and colposcopist (EFK) that conducted the exams in this *S*. *mansoni*-endemic area had performed investigations and found ample FGS in four *S*. *haematobium* endemic countries prior to this study [68]. Thus, we feel confident that, had lower genital tract lesions been present, caused by *S*. *mansoni*, we would have detected them.

In the upper genital tract, reports from pathology laboratories in *S*. *mansoni*-endemic areas have, on occasion, found *S*. *mansoni* ova and we cannot preclude that our study participants may have been affected there [58,62]. Surgeries and laparoscopies cannot be done (for ethical reasons) as a research project, however upper genital tract should be explored when laparoscopy or surgery is done for other reasons. Alternatively, lesions of the upper genital tract can be explored in connection with post-mortems.

## Conclusions

In this study population we found that *S*. *mansoni* was not associated with lower genital tract lesions or genital symptoms. However, further investigations are needed to explore the causes of genital symptoms around Lake Victoria to offer correct management. Furthermore, the possibility of lesions and pathology due to *S*. *mansoni* in the upper genital tract should be explored.

## Supporting information

S1 Table Data set for *S*. *mansoni* and genital lesions in Kenya. (XLSX)
